# Mitochondrial Dysfunction as the Major Basis of Brain Aging

**DOI:** 10.3390/biom14040402

**Published:** 2024-03-26

**Authors:** Stephen C. Bondy

**Affiliations:** 1 Department of Environmental & Occupational Health, University of California, Irvine, CA 92697, USA; scbondy@uci.edu; 2 Department of Medicine, University of California, Irvine, CA 92697, USA

**Keywords:** brain, aging, mitochondria, inflammation, excitotoxicity, oxidative stress

## Abstract

The changes in the properties of three biological events that occur with cerebral aging are discussed. These adverse changes already begin to develop early in mid-life and gradually become more pronounced with senescence. Essentially, they are reflections of the progressive decline in effectiveness of key processes, resulting in the deviation of essential biochemical trajectories to ineffective and ultimately harmful variants of these programs. The emphasis of this review is the major role played by the mitochondria in the transition of these three important processes toward more deleterious variants as brain aging proceeds. The immune system: the shift away from an efficient immune response to a more unfocused, continuing inflammatory condition. Such a state is both ineffective and harmful. Reactive oxygen species are important intracellular signaling systems. Additionally, microglial phagocytic activity utilizing short lived reactive oxygen species contribute to the removal of aberrant or dead cells and bacteria. These processes are transformed into an excessive, untargeted, and persistent generation of pro-oxidant free radicals (oxidative stress). The normal efficient neural transmission is modified to a state of undirected, chronic low-level excitatory activity. Each of these changes is characterized by the occurrence of continuous activity that is inefficient and diffused. The signal/noise ratio of several critical biological events is thus reduced as beneficial responses are gradually replaced by their impaired and deleterious variants.

## 1. Introduction

The purpose of organisms is ultimately the successful transmission of specific DNA from one generation to the next. This is true of both single-celled species and multicellular eukaryotes. Natural selection allows the appearance of species that are increasingly adapted to their environment. In animal phyla with more complex structure, the inheritance and transmission of these modifications forms the basis of Darwinian transmutation of species. Such active driving forces are only relevant during the portion of the lifespan where production and maintenance of the next generation is involved. Beyond this maturational stage, the power of trans-generational selective forces becomes irrelevant and is gradually lost. In the case of humans, medical developments have led to the lifespan often being considerably longer than the fertility span and the period of essential sustenance of offspring. After this, organisms are no longer subject to evolutionary influences. The consequent generational irrelevance means that the biology of the aged is not forced into an increasing optimal configuration. As a result, those metabolic processes, vital for organismic success are no longer subject to selective pressures and are more free to deviate from their originally tightly circumscribed limits. A more direct route taken by a specific metabolic path may increase its efficiency in isolation but not that of the organism as a whole. Such “short cuts” are prevented earlier in the life cycle when the effectiveness of the entire organism is paramount. However, in the absence of trans-generational relevance, there is nothing to prevent their increasing appearance.

This review discusses how, in the absence of selective forces, three key networks vital for defense and maintenance of nervous system integrity can become degraded and ultimately injurious rather than protective. The basis of such adverse age-related deviation is attributed in large part to diminishing competence of mitochondrial function.

## 2. The Decline of Immune System Function with Age

The continual surveillance of tissues within the body by both the circulating immune system and the immune responsivity generated intrinsically by many cell types is an important contributor to organismic survival. Such protection is by recognition and destruction of extrinsic bacteria and viruses together with endogenous abnormal or dead cells. In order to ensure effective functioning, the elimination of undesirable materials has to take place in a focused manner, with a minimal penumbra that would involve proximal healthy cells. This system develops during maturation when the protective effect of maternal antibodies in the young progressively decreases. Innate immunity depends on neutrophils and macrophages, and these proliferate shortly after birth. Adaptive immunity requires the development of appropriate antibodies for a range of antigens. Both T cells and B cells require maturation and also experience, in order to become fully functional by allowing T cells to present antigens to B cells which can then produce specifically directed antibodies. This developmental process is promoted by some desirable constituents of the intestinal microbiome, notably *Bifidobacterium infantis* [1].

Several autoimmune disorders can develop early in life, or in adulthood with earlier onset generally being associated with elevated disease severity [2]. These include such diseases as type 1 diabetes and asthma, multiple sclerosis, and rheumatoid arthritis. These diseases have a significant genetic component, but environmental factors also determine whether and when the disease will be expressed. 

A different type of inflammatory disturbance is found in many age-related diseases. This consists of a low level of sustained systemic inflammation which, unlike a targeted immune response, this inflammation is independent of any provocative stimulus. Such a chronic condition has a wide variety of adverse effects and plays a significant role in the pathology of a wide range of diseases whose prevalence rises with aging. These include ischemic heart disease, vascular disease, cancer, Type II diabetes, and fatty liver disease. Accelerated appearance of the condition has been attributed to many underlying dietary and lifestyle factors [3]. Aging is likely to be a key but not an exclusive factor. Such extended inflammation forms a significant component of nearly all neurodegenerative disorders including Alzheimer’s disease, Parkinson’s disease, Huntington’s disease, and amyotrophic lateral sclerosis [4,5]. Neuroinflammation may well be a primary mechanism of Alzheimer’s disease rather than a secondary consequence [6] and may be the factor that drives the formation of tangled tau proteins and amyloid plaque [7]. This age-related malfunction seems attributable to both innate and adaptive immune mechanisms [8].

## 3. The Excessive Presence of Oxidant Free Radicals with Aging

Hydrogen peroxide (H_2_O_2_),the superoxide anion radical (O_2_−), and nitric oxide (NO.) are key signaling agents. They are generated by many enzymes notably by the mitochondrial respiratory chain. Redox signaling is a critical mechanism in the regulation of many metabolic processes and adaptive responses to various stressors [9]. This signaling is especially prominent in the brain due to its high energy demand [10,11]. Any excess content or diffusion of these more stable precursors of highly reactive short-lived oxidant free radicals can be effectively regulated by the presence of the several antioxidant enzymes, such as catalase, superoxide dismutase and peroxidases.

With senescence, the efficiency of underlying processes is diminished, and a redox imbalance emerges between the synthesis and degradation of these species by antioxidant enzymes and chemicals [12]. The failure of homeostasis leads to free radical-induced damage to macromolecules. This has been linked to the aging process and also to the emergence of many age-associated disease classes including neurodegenerative disease, cardiovascular disease and cancer [13]. The issue of whether oxidative stress is the primary cause or secondary consequence of the disease remains unclarified.

## 4. The Emergence of a Chronic State of Low-Level Excitatory Activity

The aging brain is characterized by a minor but permanent presence of undirected hyperexcitation. The basis for this includes heightened content of free calcium ions within the cell which can be attributed to failure of their mitochondrial sequestration. This results in an excessive level of calcium-effected neurotransmitter release and overstimulation of glutaminergic NMDA receptors. The prolonged activation of glutamate receptors leads to enhanced calcium influx, further mitochondrial failure, and oxidative stress [14]. In addition to initiating excitotoxicity, excess intracellular calcium causes the activation of calpain [15]. This protease can disrupt intracellular architecture, leading to the emergence of a range of metabolic defects and ultimately to apoptosis. Calpain also contributes to the emergence of several inflammatory events including activation of NF-kB and inflammatory cytokines. Calpains participate in many age-related non-resolving inflammatory diseases, including atherosclerosis and rheumatoid arthritis [16]. Calpain can affect the activation of astrocytes from resting to the reactive fibrous astrocytes and can increase microglial reactivity. These chronic neuroinflammatory responses lead to neuronal loss [17]. Inhibition of calpain diminishes microglial and astroglial responsivity and reduces the extent of the neuropathological changes and behavioral decline of aged transgenic mice modeling Alzheimer’s disease [18,19,20] and Parkinson’s disease [21,22]. Glutamate receptors that respond to excess activation are also found in oligodendrocytes which can be damaged by excitotoxic events [23]. NMDA receptor stimulation also induces activation of NADPH oxidase 2, a magnitude relation enzyme that leads to production of reactive oxygen species which can harm surrounding cells [24]. The transition from the ability of activation of the NMDA receptor as a means to initiate plasticity and stimulate cell survival, to such activation leading to advancement of cell death in Alzheimer’s disease, may reside in the shift of this receptor shift from a germane intrasynaptic location to irrelevant extrasynaptic sites [25]. The use of NMDA antagonists may protect against age-related neurodegenerative disease [26].

## 5. The Mitochondrial Basis for Age-Related Deviation of Essential Processes toward Injurious Configurations

The inevitability of the distortion of the three key metabolic processes described must ultimately be ascribed to failure of repair and maintenance programs that were previously functional. There is evidence that most of these deficits are triggered by the onset and accumulation of mitochondrial flaws during senescence. Effective mitochondrial functioning is vital for organismic function. Mitochondrial quality, which is a prime determining factor in the health of cerebral cell, declines with aging and even more so in neurodegenerative disease [27]. The three adverse transformations described above are all found in aging mitochondria and this organelle may trigger effects throughout the entire cell. Mitochondrial dynamics of fusion, fission and mitophagy are reduced with age, and the consequent reduction of mitochondrial quality can lead to a series of adverse consequences [28].

A wide range of changes can be observed in the mitochondria of aged subjects. These include greater leakage of free radicals during oxidative phosphorylation and an accumulation of mutations in mtDNA [29]. A higher rate of single point mutations in mtDNA has been found in brains from the elderly in comparison with those from the young [30]. These mutations often involve deletion of DNA and consequent failure of productive oxidative phosphorylation [31]. Such ineffective mitochondria can be clonally amplified along with healthy mitochondria. In younger healthy animals, mitochondrial dynamics of division and fusion serve to minimize the accumulation of such unproductive organelles, but the efficiency of this process is diminished with age [28]. 

The harmful changes in mitochondria that are emphasized with age, involves not only deficits in mtDNA replication but also release of nuclear chromatin fragments into the cytoplasm induced by mitochondrial factors [32]. These changes lead to a hypometabolic state in the aged brain [10]. In a genetic mouse model where mitochondrial polymerase-γ is blocked, premature aging occurs, and longevity is drastically reduced [33].

### 5.1. Free Radical Production

Deficits in mitochondrial ATP production are accompanied by elevated leakage of reactive oxygen species as aging proceeds [34]. ROS are components of key intracellular signaling systems that can modulate the activity of several Ca^2+^ channels [35].

Paradoxically, there is also contrary evidence that indicates that a mild diminution of mitochondrial effectiveness can result in lengthening of the lifespan. Since this effect is blocked by antioxidants, it seems that this effect is due to the hormetic effect of low levels of reactive oxygen species [28,36]. Moderate activation of stress responses may restore the effectiveness of clearance of improperly folded proteins that accumulate with aging [37]. 

Potentially reparable mitochondrial changes include reversal of transient mtDNA deletions that reduce the effective functioning of oxidative phosphorylation. Permanent loss of the intact and functional mitochondrial genome following oxidative stress may be due to the excessive and harmful prolongation of processes that could efficiently repair mtDNA in response to transient moderate stressors. Prolonged excess production of reactive oxygen species leads to irreversible mitochondrial damage rather than leading to a reversible and useful adaptation [38]. This dysregulation is likely to grow with age where chronic stressors tend to accumulate [39].

### 5.2. Undesirable Autoimmune Responses

Unlike nuclear DNA, mitochondrial DNA is not methylated. Since unmethylated DNA is also found in bacteria, the appearance of mtDNA in the cytoplasm can invoke an autoimmune response by way of the innate immune system [36]. mtDNA can be sensed by several moieties including Toll-like receptor 9, and the NLRP3 inflammasome. When activated, all these will lead to the production of inflammatory cytokines [40]. 

The mtDNA fragments released by damaged aging mitochondria contain bacterial-like CpG segments [41]. These are recognized and interpreted as a threat by the innate immune system, and a response is triggered by way of the cGAS-STING signaling pathway, a regulator of senescence-associated secretory phenotype (SASP). Cyclic GMP-AMP synthase (cGAS) recognizes cytoplasmic DNA fragments and this effects the stimulation of interferon genes (STING) [42]. This in turn promotes formation of inflammatory cytokines such as TNF-a. Such untargeted inflammation leads to neurodegeneration [43]. The leakage of mitochondrial DNA into the cytosol may thus be a key factor in accounting for many age-related diseases as being based on autoimmune responses. Such a condition is typical of cells that have lost functionality but have evaded the apoptotic pathway [44]. Inhibition of excessive mitochondrial permeability in aged mice decreases the escape of mtDNA fragments and calcium into the cytosol and this leads to reduction of inflammation and reduces the progression of markers of senescence [45]. 

### 5.3. Persistent Hyperexcitation

Following electrical activity, the cytosolic calcium level is increased as the wave of axonal depolarization arrives at the synapse. The mitochondrion rapidly takes up this excess calcium by way of the calcium uniporter complex. This prevents further calcium-initiated repetitive transmitter release. Calcium can then be more gradually released into the cytosol and thence out of the cell by Na+/Ca^2+^ and H+/Ca^2+^ exchangers. By this means, the mitochondrion is critical for optimal regulation of synaptic transmission. Oxidative stress can be a secondary consequence of mitochondrial calcium overload in excitotoxicity [46,47] and can promote the opening of the non-selective mitochondrial permeability transition pore (mPTP). This can lead to mitochondrial swelling, reduced capacity for oxidative phosphorylation and ultimately, apoptic cell death. Such changes are prominent in aging but causality is difficult to pinpoint due to the reciprocal interactions between these systems. Most of the energy requirements of the brain are concerned with maintenance of ion gradients and their rebuilding after neural activity. Diminution of ATP production by aged mitochondria can lead to increased glutamatergic excitotoxicity [48]. It is noteworthy that synapses with few mitochondria in their vicinity exhibit hyperexcitability [49].

Several age-related neurodegenerative disorders are characterized by a common feature, namely excessive calcium levels within mitochondria which inevitably leads to heightened cytoplasmic calcium content and thence to elevated glutamatergic activity [50]. Rather than resulting in overt excitotoxicity, this lesser level of rising cytosolic calcium may lead to a prolonged extension of excitatory postsynaptic potentials [49]. Failure of mitochondrial calcium buffering may have similar deficits leading to excitotoxicity in normal aging [48]. 

## 6. Importance of Mitophagy

A major factor in maintaining the quality of mitochondria is the clearance of non-functioning variants by mitophagy. However, the vigor of mitophagy is reduced as the brain ages. An important aspect of cellular quality control is removal of ineffective mitochondria by mitophagy. However, the intensity of mitophagy is reduced with brain aging. Both mitogenesis and phagocytic removal of aberrant mitochondria are reduced with age [51]. 

The cellular content of ineffective mitochondria is gradually increased for several reasons. Firstly, while DNA deletions can cause loss of genes required for oxidative phosphorylation, such diminution in size may enable faster replication than that of normal mitochondria. 

Secondly, there is evidence that mitochondria can be transferred between cells of the nervous system [52]. This could allow dissemination of flawed mitochondria and induction of an inflammatory response, thereby contributing to neurodegeneration [53]. Alternatively, damaged mitochondria can be transferred from neurons to astrocytes where they can be effectively degraded [22]. Reduced mitophagy may lead to an excessive content of defective mitochondria in AD. Depression in the levels of glucose consumption characterize cerebral senescence. Age-related mitochondrial dysfunction may be an early component of the development of AD, predating the onset of clinical symptoms [54]. 

**Figure 1:**
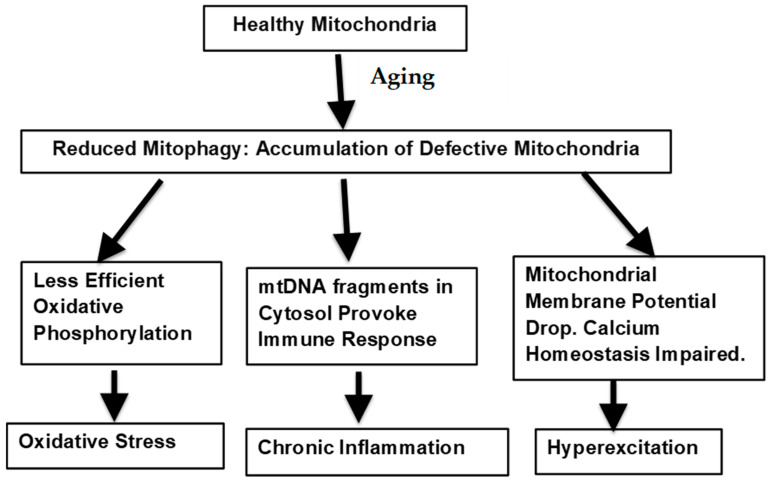
Mitochondrial factors underlying neurological aging.

The potential means by which defective mitophagy could lead to the age-related warping of normal biological processes is illustrated in Figure 1. 

## 7. Therapeutic Moderation of the Rate of Aging

Effective mitophagy has been associated with longevity in several species [55]. In a variety of experimental systems, stimulation of mitophagy has been found to promote overall organismic health, extend the lifespan and retard cognitive decline [56]. Human trials are currently in progress [57,58]. The mitochondrion may thus be a suitable target for developing new therapeutic strategies for treatment not only of AD but for slowing brain aging in general [59]. A wide range of bioactive dietary constituents may stimulate mitophagy within the brain. Dietary agents that may enhance mitogenesis in the brain include resveratrol, spermidine, and curcumin, all of which are likely to act by way of the SIRT1 pathway [60].

While the benefits of most of these agents have been ascribed to their antioxidant properties, the use of broad-spectrum antioxidants has not been demonstrated to effectively extend the lifespan or retard the onset rate of cognitive or neurological evidence of senescence [61]. However, the application of antioxidants specifically targeting the mitochondrion is a promising means of effecting a more selective protection of these organelles. This could be achieved by linkage of antioxidants to lipophilic cations including lipophilic peptides, or by use of liposomally encapsulated antioxidants [62]. All of these modifications are designed to enhance the ability of antioxidants to improve access of antioxidants by allowing them to penetrate the charged mitochondrial outer membrane. The ability of these micronutrients to promote degradation of ineffective mitochondria may also involve more specific mechanisms by way of the stress-responsive neurotrophic factor BDNF, mTOR and the sirtuin pathway. In addition, mitogenesis can also be enhanced by mitohormesis, namely application of mild stressors such as exercise and hypoxia by way of the hypoxia-induced mitogenic factor [17], or oxidative stress by way of the Nrf2 signaling pathway [60,63,64,65]. Equally important is the clearance of aged ineffective mitochondria. Several of the micronutrients and exercise strategies that simulate mitogenesis have also been reported to enhance mitophagy [57]. Similarly, mitochondrial turnover is improved by caloric restriction [66]. The processes of synthesis and elimination of mitochondria appear to be linked. With brain aging and more so with neurodegenerative disease, the normal cycle of elimination and regeneration is disrupted and slowed [57]. 

## 8. Conclusions

All of the systems described above are interrelated, and deficits in one can rapidly have a reciprocal impact on others. Declining energy production by less efficient mitochondria disrupts Ca^2+^ buffering which leads to excitotoxicity and enhances generation of reactive oxygen species. Excess ROS leads to increased accretion of mutations in mtDNA [35]. Fragments of mtDNA entering the cytosol can then provoke inflammatory responses. The role of the mitochondrion in being causal in the deformation of several vital intracellular activities is summarized in Table 1.

The key metabolic operations described initially, which are essentially adaptive in nature, and their competence is generally maintained through maturity but inevitably begin to decline with senescence. The reason for this broad-based failure may relate to the evolutionary forces driving optimal metabolic efficiency. Presumably these are in effect for around the first forty years of human life when reproduction and nurturing of offspring are cardinal. The maintenance of the best survival strategies over this time period may be at the expense of later biological competence. The lack of evolutionary pressure in later life may account for the onset of neurodegenerative disorders due to the absence of corrective imperatives. The deficits discussed all involve a falling off of the proportion of a meaningful signal relative to irrelevant background activity. The failure of effective mitochondrial function may underlie all three of the age-related modulations described and may lead to the onset of several neurodegenerative diseases [67]. The basis for such progressive failure may relate to the lack of a regulating evolutionary track-line after mid-life. Any significant deficits occurring prior to this would be subject to strict pruning by evolutionary forces. In the absence of such focusing imperatives, a certain drift takes place. Receptors diffuse from their functional sites to extraneous locations where they act in a less directed manner that is often harmful. Rather than accurately targeting invasive species or abnormal cells, the immune system maintains a more dispersed but less purposeful presence, leading to autoimmune incursions. There is increased production of reactive oxygen species which can be randomly destructive, consequent to less efficient metabolic transformations. 

The mitochondrion is active throughout life and undergoes many replications. The mitochondrial genome has 100-fold higher mutation rate than the nuclear genome due to replication and repair errors and the absence of protective histones. This is due to the mitochondria being the site of high levels of reactive oxygen species due to leakage from the electron transport respiratory chain, causing single- or double-strand breakage of mtDNA. The excision process that repairs oxidized mitochondrial mtDNA bases is subject to an age-related decline in the brain, and this is exacerbated in the case of several age-related neurological diseases [68]. In addition, the fidelity of mitochondrial DNA polymerase is much lower than that of nuclear DNA polymerases and can further contribute to the heteroplasmy of mtDNA [69]. Any independent evolutionary appearance of less competent variants will be curtailed in the young where the pressure of natural selection is borne by the entire organism. With age this selection for efficiency of the whole eukaryote dissipates, leading to greater mtDNA diversity [70]. Combined with the large number of replications undertaken by the mitochondria, this implies that these organelles are subject to intense competitive influences within the cell. This allows mitochondrial variants free rein for contest among themselves with the most rapidly dividing variants gaining a selective advantage. However, such pressures made in the best interest of individual mitochondrial survival may not always align with the best interests of the whole organism. However, while this drift may be unavoidable, there are several means by which the rate of drift toward the age-related appearance of inoperative mitochondria may be delayed.

## Figures and Tables

**Table 1:** Mitochondrial basis of age-related derangement of essential neurobiological processes.

Essential Activity	Ineffective Potentially Harmful Variant	Means by Which Mitochondria can Contribute to This Transition
Generation of energy by oxidation of substrates	Leakage of harmful free radicals into the cytosol	Decreased efficiency of the mitochondrial oxidative phosphorylation
Dispersion of invasive organisms by oxidant free radicals	Balance between antioxidant molecules and reactive oxygen disrupted	Sub-optimal redox status leads to failure of Mitochondrial Unfolded Protein Response (mtUPR) and misfolding of proteins
Removal of invasive organisms and unhealthy cells by glial and phagocytic immune attack	Extended untargeted inflammatory activity leading to random cell injury	DNA from impaired mitochondria leaks into cytosol provoking autoinflammatory response
Maintenance of neuronal signaling	Persistent unfocussed neuronal excitatory activity	Failure of mitochondria to sequester calcium and activation of calpains leads to increased intrasynaptic glutamate release.
